# Rottlerin Inhibits *Lonicera japonica*-Induced Photokilling in Human Lung Cancer Cells through Cytoskeleton-Related Signaling Cascade

**DOI:** 10.1155/2011/193842

**Published:** 2011-02-08

**Authors:** Bang-Jau You, Yang-Chang Wu, Bo-Ying Bao, Chi-Yu Wu, Ya-Win Yang, Yu-Hao Chang, Hong-Zin Lee

**Affiliations:** ^1^School of Chinese Medicine Resources, China Medical University, 91, Hsueh-Shih Road, Taichung 40402, Taiwan; ^2^Graduate Institute of Integrated Medicine, China Medical University, 91, Hsueh-Shih Road, Taichung 40402, Taiwan; ^3^School of Pharmacy, China Medical University, 91, Hsueh-Shih Road, Taichung 40402, Taiwan; ^4^Department of Internal Medicine, Chang-Hua Hospital, 80, Chung-Cheng Road Sec. 2, Puhsin 51341, Chang-Hua, Taiwan

## Abstract

This study demonstrated that many apoptotic signaling pathways, such as Rho family, PKC family, MAP kinase family, and mitochondria-mediated apoptotic pathway, were triggered by *Lonicera japonica* extracts and irradiation in CH27 cells. Rottlerin, a PKC**δ** -selective inhibitor, reversed the photoactivated *Lonicera japonica* extract-induced decrease in PKC**δ** protein expression and change in cell morphology in this study. In addition, rottlerin inhibited the photoactivated *Lonicera japonica*-induced decrease in protein expression of Ras, ERK, p38, PKC**α**, and PKC**ε**, which are the kinases of prosurvival signaling pathway. We also demonstrated that pretreatment with rottlerin prevented actin microfilaments and microtubules from damage during the photoactivated *Lonicera japonica*-induced CH27 cell death. Furthermore, the promotion of the cytoskeleton-related signaling cascade following rottlerin by upregulation of cytoskeleton-related mediators (p38, HSP27, FAK, paxillin, and tubulin) and molecules of downstream of F-actin (mitochondria-mediated apoptosis pathway) reduces CH27 cell death, indicating that cytoskeleton is the potential target in the photoactivated *Lonicera japonica*extract-induced photokilling of CH27 cells.

## 1. Introduction

Photodynamic therapy is becoming widely accepted as a potential treatment for many forms of cancer and based on the administration of a tumor-localizing photosensitizing agent which is activated by light of appropriate wavelength. It has been demonstrated that photodynamic therapy is a treatment option for lung cancer that involves the administration of a photosensitizing agent and fiberoptic bronchoscope, which was used to deliver of light to tumor tissue that has retained the agent [1, 2]. Our previous study demonstrated that *Lonicera japonica* extracts exhibited significant photocytotoxicity in human lung squamous carcinoma CH27 cells at a concentration range of 50–150 *μ*g/mL, with 0.4–1.2 J/cm^2^ light dose [3]. The p38-associated pathway was demonstrated to be involved in the photoactivated* Lonicera japonica* extract-induced CH27 cell apoptosis [3]. 

Apoptosis is a major form of cell death, which involves many factors and signal transduction molecules. The severe morphological changes in apoptotic cells were demonstrated that apoptosis has dramatic implications on the cytoskeleton. Several reports suggested that the actin cytoskeleton plays an important role in the early stage of inducing of apoptosis [4, 5]. Many investigators also indicated that mitochondrial activity as reflected by mitochondria membrane potential should have a significant bearing for F-actin assembly [6, 7]. Therefore, this study examined the relationship between the redistribution of F-actin and mitochondrial function in the photoactivated *Lonicera japonica* extract-induced CH27 cell death. 

Rottlerin, also called mallotoxin, is a natural compound isolated from the tree *Mallotus philippinensis* (the monkey-faced tree). Many studies have employed rottlerin as a protein kinase C *δ*-selective inhibitor and mitochondrial uncoupler [8–11]. Therefore, rottlerin has both proapoptotic and antiapoptotic effects, which are consistent with an anti-apoptotic effect of PKC*δ* and rottlerin acting via uncoupling mitochondria, because classical mitochondrial uncouplers can promote apoptosis [9–11]. In this study, we found that rottlerin blocked the photoactivated *Lonicera japonica* extract-induced CH27 cell death. Therefore, we used rottlerin to investigate the mechanisms of photoactivated *Lonicera japonica* extract-induced CH27 cell death.

We examined the effect of *Lonicera japonica* extracts with light on cytotoxicity in several tumor cell lines. A series of photocytotoxic screen tests showed that *Lonicera japonica* extracts with light exhibited significant cytotoxicity in lung carcinoma cells (CH27 and H460), skin cancer cells (M21), and oral cancer cells (HSC-3). The overall objective of the present study is to further explore the integrated mechanism of photoactivated *Lonicera japonica*-induced human lung carcinoma CH27 cell apoptosis. We also used rottlerin as a tool of research to demonstrate that cytoskeleton is the potential target in photoactivated *Lonicera japonica* extract-induced CH27 cell apoptosis.

## 2. Methods

### 2.1. Materials

The voucher specimens (*Lonicera japonica*: CMU LJ 0614) were deposited in School of Chinese Medicine Resources, China Medical University, Taichung, Taiwan. Antipain, aprotinin, dithiothreitol (DTT), ethyleneglycol-bis(*β*-aminoethyl ether)-N,N,N′,N′-tetraacetic acid (EGTA), leupeptin, pepstatin, phenylmethylsulfonyl fluoride, rottlerin (1-[6-[(3-acetyl-2,4,6-trihydroxy-5-methylphenyl)methyl]-5,7-dihydroxy-2,2-dimethyl-2H-1-benzopyran-8-yl]-3-phenyl-2-propen-1-one), Tris, and TRITC (tetramethylrhodamine isothiocyanate)-conjugated phalloidin were purchased from Sigma Chemical Company (St. Louis, MO, USA). FAK, *β*-actin, and HSP27 antibodies were from Sigma Chemical Company. Ras, Rho, Cdc42, ERK, JNK, p38, PKC*α*, PKC*δ*, and PKC*ε* antibodies were purchased from BD Biosciences (San Diego, CA, USA). Tubulin and paxillin antibodies were purchased from Calbiochem (San Diego, CA, USA).

### 2.2. Preparation of *Lonicera japonica*

The air-dried plants of *Lonicera japonica* (200 g) were soaked three times with 1 L of 95% ethanol at room temperature for 3 days. The extracts were filtered. The filtrates were collected and then concentrated under reduced pressure at 40°C. The yield of dry extract of *Lonicera japonica* was about 11%.

### 2.3. Cell Culture

CH27 cells were grown in monolayer culture in Dulbecco's modified Eagle's medium (DMEM; Gibco BRL, Rockville, MD, USA) containing 10% FBS (HyClone, Logan, UT, USA), 100 U/mL penicillin and 100 *μ*g/mL streptomycin (Gibco BRL), and 2 mM glutamine (Merck, Darmstadt, Germany) at 37°C in a humidified atmosphere comprised of 95% air and 5% CO_2_. When CH27 cells were treated with *Lonicera japonica* extracts, the culture medium containing 1% FBS was used.

### 2.4. Light Source

The irradiation source was a set of fluorescent lamp (20 W, China Electric MFG. Corporation, Taiwan) located at a made-to-measure box. The wavelength of fluorescence lamp is in the range of 400–700 nm. The intensity of light was measured as Lux, a system international illumination measure. Lux was inverted to light dose (J/cm^2^). The cells were irradiated at 40 W for 30 min that correspond to 0.8 J/cm^2^ light dose.

### 2.5. Protein Preparation

Cells were seeded at a density of 1.7 × 10^6^ cells onto 10-cm dish 48 h before being treated with drugs. CH27 cells were incubated with 100 *μ*g/mL* Lonicera japonica* extracts for 4 h and then irradiated with 0.8 J/cm^2^ fluence dose. After irradiation, adherent and floating cells were collected at the indicated time intervals and washed twice in ice-cold phosphate-buffered saline (PBS). Cell pellets were resuspended in lysis buffer (50 mM Tris-HCl, pH 7.5, 150 mM NaCl, 1% Nonidet P-40, 0.25% sodium deoxycholate, 1 mM EGTA, 1 mM DTT, 1 mM phenylmethylsulfonyl fluoride, 1 mM sodium orthovanadate, 1 mM sodium fluoride, 5 *μ*g/mL aprotinin, 5 *μ*g/mL leupeptin, and 5 *μ*g/mL antipain) for 30 min. Lysates were clarified by centrifugation at 13,000 rpm for 30 min at 4°C and the resulting supernatant was collected, aliquoted, and stored at −80°C until assay. The protein concentrations were estimated with the Bradford method.

### 2.6. Western Blot Analysis

Samples were separated by various indicated concentrations of sodium dodecyl sulfate-polyacrylamide gel electrophoresis (SDS-PAGE; Bio-Rad, Hercules, CA, USA). The SDS-separated proteins were equilibrated in transfer buffer (50 mM Tris-HCl, pH 9.0–9.4, 40 mM glycine, 0.375% SDS and 20% methanol) and electrotransferred to Immobilon-P Transfer Membranes (Millipore Corporation, Bedford, MA, USA). The blot was blocked with a solution containing 5% nonfat dry milk in Tris-buffered saline (10 mM Tris-HCl and 150 mM NaCl) with 0.05% Tween 20 (TBST) for 1 h, washed, and incubated with various indicated antibodies. Secondary antibody consisted of a 1 : 20,000 dilution of horseradish peroxidase (HRP)-conjugated goat antimouse IgG (for PKC*α*, Ras, Rho, Cdc42, ERK, JNK, p38, tubulin, and paxillin) or antirabbit IgG (for PKC*δ*, PKC*ε*, FAK, and HSP27). The enhanced chemiluminescent (NEN Life Science Products, Boston, MA, USA) detection system was used for immunoblot protein detection.

### 2.7. Mitochondrial Reductase Activity

Cells were seeded at a density of 1 × 10^5^ cells per well onto a 12-well plate 48 h before being treated with drugs. The cells were incubated with 0.1% ethanol or 100 *μ*g/mL* Lonicera japonica* extracts for 4 h and then irradiated with 0.8 J/cm^2^ fluence dose. After irradiation, the cells were washed with PBS. Cellular mitochondrial reductase activity of live CH27 cells was determined by measuring the reduction of 3-(4,5-dimethylthiazol-2-yl)-2,5-diphenyltetrazolium bromide (MTT). At each end point, the treatment medium was replaced with fresh serum-free medium containing 2.4 × 10^−4^ M MTT at pH 7.4. Cells were incubated with MTT medium for 1 h at 37°C. After solubilization in dimethylsulfoxide, absorbance was measured at 550 nm.

### 2.8. Morphological Investigation

Cells were seeded at a density of 1 × 10^5^ cells per well onto 12-well plate 48 h before being treated with drugs. The cells were incubated with 0.1% ethanol or 100 *μ*g/mL* Lonicera japonica* extracts for 4 h and then irradiated with 0.8 J/cm^2^ fluence dose. After irradiation, the cells were photographed immediately with an Olympus IX 70 phase-contrast microscopy (300X). A field was chosen in the center of each well at approximately the same location for photography.

### 2.9. Localization of F-Actin and Tubulin

Qualitative F-actin and tubulin staining was obtained by plating CH27 cells at 1 × 10^5^ cells per well 48 h. CH27 cells were incubated with vehicle alone or 100 *μ*g/mL *Lonicera japonica* extracts for 4 h and then irradiated with 0.8 J/cm^2^ fluence dose. To visualize F-actin, cells were fixed in 3.7% formaldehyde for 15 min, permeabilized with 1% Triton X-100 for 10 min, and incubated with 1.9 × 10^−7^ M TRITC-phalloidin for 40 min. To detect tubulin, cells were incubated for 30 min with 250 nM Tubulin Tracker Green reagent. After three washings, the cells were observed by Olympus IX 70 fluorescence microscopy (300X).

### 2.10. Measurement of Mitochondrial Membrane Potential

Mitochondrial membrane potential (MMP) was measured with JC-1 (5,5′,6,6′-tetrachloro-1,1′,3,3′-tetraethylbenzimidazolocarbocyanine iodide; Molecular Probe, Eugene, OR). CH27 cells (1 × 10^6^) were incubated with vehicle alone or 100 *μ*g/mL of *Lonicera japonica* extracts for 4 h and then irradiated with 0.8 J/cm^2^ light dose. After irradiation, cells were stained with 2 *μ*M JC-1 for 30 min, washed with PBS, and analyzed on a flow cytometer (Becton Dickinson, San Joes, CA, USA) using 488 nm excitation with 530 and 590 nm bandpass emission filters.

### 2.11. Measurement of Mitochondrial Permeability Transition (MPT) Pore with Calcein

Cells were seeded at a density of 6 × 10^5^ cells onto 6-cm dish 48 h before being treated with drugs. CH27 cells were loaded with 1 *μ*M calcein-acetomethoxy ester for 30 min in 1 mL of DMEM supplemented with 1 mM CoCl_2_. Cells were washed free of calcein and incubated with vehicle alone or 100 *μ*g/mL* Lonicera japonica* extracts for 4 h and then irradiated with 0.8 J/cm^2^ fluence dose. After irradiation, the fluorescence intensity of calcein was measured with FACSCanto flow cytometer (excitation, 488 nm; emission, 530 nm; Becton Dickinson) and analyzed using ModFit LT 3.0 Software (Verity Software House, Topsham, ME, USA).

### 2.12. Statistical Analysis

Standard statistical methods based on Student's *t*-test and regression analysis were used. The results are expressed as percentage ±S.D. of control.

## 3. Results

### 3.1. Effects of Photoactivated *Lonicera japonica* Extracts on the Protein Expression of Anti- and Proapoptotic Members in CH27 Cells

**Figure 1:**
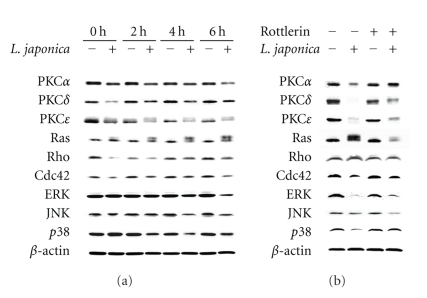
(a) The effect of photoactivated *Lonicera japonica* extracts on the protein expression of PKC, Rho and MAP kinase family members in CH27 cells. Cells were incubated with 0.1% ethanol or 100 *μ*g/mL* Lonicera japonica* extracts for 0, 2, 4, and 6 h and then irradiated with 0.8 J/cm^2^ fluence dose. (b) Effects of rottlerin on the photoactivated *Lonicera japonica* extract-induced changes in the protein expression of PKC, Rho, and MAP kinase family members in CH27 cells. Cells were pretreated with 10 *μ*M rottlerin for 1 h and then treated with 0.1% ethanol or* Lonicera japonica* extracts (100 *μ*g/mL, 4 h) and light (0.8 J/cm^2^). After irradiation, cell lysates were analyzed by 7% (PKC*α*, *δ* and *ε*), 12% (ERK, JNK and p38) and 14% (Ras, Rho and Cdc42) SDS-PAGE and then probed with primary antibodies as described in Section 2. −: control cells; +: *Lonicera japonica* extract- or rottlerin-treated cells. Results are representative of three independent experiments.

To investigate the role of anti- or proapoptotic members in modulating cell apoptosis induced by photoactivated *Lonicera japonica* extracts, this study detected the protein expression of PKC, Rho, and MAP kinase family members by Western blot analysis. After treatment of CH27 cells with 100 *μ*g/mL *Lonicera japonica* extracts and 0.8 J/cm^2^ light dose, the expression of PKC*α*, PKC*δ*, PKC*ε*, Ras, Rho, Cdc42, ERK, JNK, and p38 proteins significantly decreased in photoactivated *Lonicera japonica* extract-treated cells within 6 h (Figure 1(a)). As shown in Figure 1(a), treatment of cells with 0.1% ethanol and light did not affect the protein expression. It is noteworthy that the protein expression of Ras exhibits two bands (21 and 22 kDa). The amount of the band of 22 kDa significantly increased after treatment with *Lonicera japonica* extracts and light (Figure 1(a)).

### 3.2. Effects of Rottlerin on the Photoactivated *Lonicera japonica* Extract-Induced CH27 Cell Death

**Figure 2:**
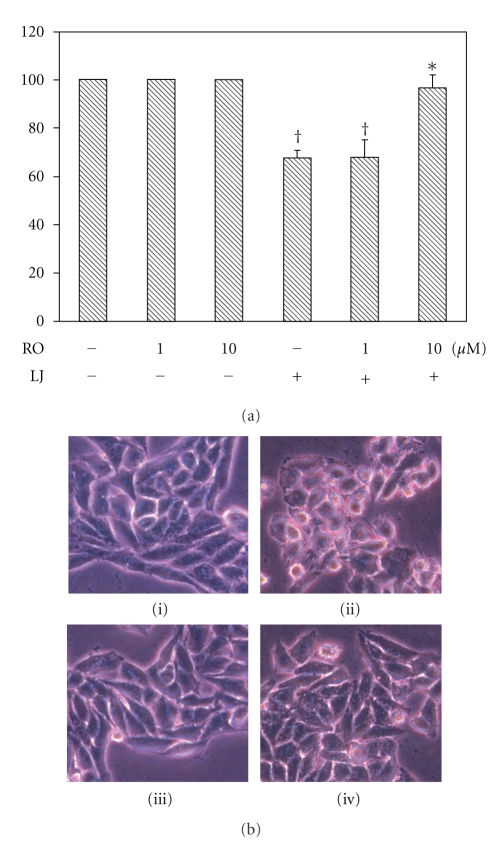
Effects of rottlerin on the photoactivated *Lonicera japonica* extract-induced cell death and changes in cell morphology. Cells were pretreated with 10 *μ*M rottlerin (RO) for 1 h and then treated with 0.1% ethanol or 100 *μ*g/mL* Lonicera japonica *extracts (LJ) for 4 h and 0.8 J/cm^2^ light dose. (a) After irradiation, the viable cells were measured by MTT assay. Results are expressed as the mean percentage of control ± S.D. ^†^ *P* < .01 compared to the control values. *P < .01 compared to the photoactivated* Lonicera japonica* extract*-*treated cells. (b) Morphological analysis by phase-contrast microscopy of CH27 cells. After irradiation, the cells were immediately photographed. (i) control cells; (ii)* Lonicera japonica*-treated cells; (iii) rottlerin-treated cells; (iv) cells were pretreated with rottlerin and then *Lonicera japonica.* All results are representative of three independent experiments.

These above data indicated that the protein expression of PKC*δ* was involved in photoactivated *Lonicera japonica* extract-induced cell death. However, PKC*δ* cleavage and activation has been reported as a proapoptotic response to various apoptotic stimuli. To further investigate whether CH27 cell death induced by treatment with *Lonicera japonica* extracts and light could be linked to the activation of PKC*δ* expression, we determined the effect of PKC*δ*-selective inhibitor rottlerin on the photoactivated* Lonicera japonica* extract-induced CH27 cell death. As shown in Figure 2(a), the photoactivated *Lonicera japonica* extract-induced CH27 cell death was blocked by pretreatment with 10 *μ*M rottlerin for 1 h. Furthermore, pretreatment of CH27 cells with 10 *μ*M rottlerin could inhibit the photoactivated *Lonicera japonica* extract-induced changes in cell morphology (Figure 2(b)). In light-shield condition, *Lonicera japonica* extracts (100 *μ*g/mL, 4 h) did not exhibit significant cell toxicity following MTT assay (data not shown). Light alone did not affect cell survival in this study (Figure 2(b)). We also demonstrated that rottlerin significantly reversed the photoactivated *Lonicera japonica* extract-mediated decrease in protein expression of PKC*α*, PKC*δ*, PKC*ε*, ERK, and p38 (Figure 1(b)).

### 3.3. Effects of Rottlerin on the Photoactivated *Lonicera japonica* Extract-Induced Changes in the Localization of Cytoskeleton in CH27 Cells

**Figure 3:**
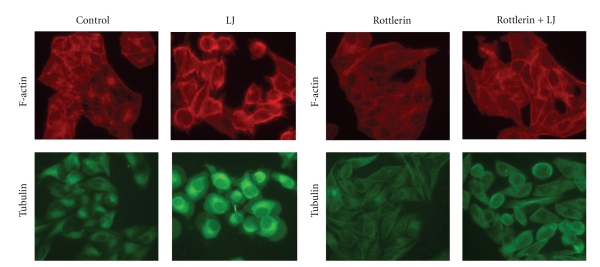
Effects of rottlerin on the photoactivated *Lonicera japonica* extract-induced changes in cytoskeleton in CH27 cells. Cells were incubated with 0.1% ethanol or 100 *μ*g/mL* Lonicera japonica* extracts (LJ) for 4 h and then irradiated with 0.8 J/cm^2^ fluence dose. In rottlerin treatment, cells were pretreated with a final concentration of 10 *μ*M rottlerin for 1 h. F-actin microfilaments in CH27 cells was visualized by TRITC-labeled phalloidin binding. To detect tubulin distribution, cells were incubated for 30 min with 250 nM Tubulin Tracker Green reagent. The specimens were observed by fluorescence microscopy (300X). Results are representative of three independent experiments.

In this study, the effect of rottlerin on the photoactivated *Lonicera japonica* extract-induced changes in CH27 cell actin microfilaments was examined. As shown in Figure 3, the photoactivated *Lonicera japonica* extract-induced disruption of actin microfilaments was prevented by pretreatment of CH27 cells with 10 *μ*M rottlerin. In the control, actin microfilaments were diffusely present as short microfilaments throughout the CH27 cells (Figure 3). Rottlerin-treated cells had a similar appearance to those in the control (Figure 3). In addition to actin microfilaments, microtubules are widely recognized as key components of the cytoskeleton. To further examine whether the microtubules were injured by photoactivated *Lonicera japonica* extracts in CH27 cells, TubulinTracker Green reagent, which is highly selective for polymerized tubulin, was used. In this study, tubulin organized into discrete aggregates dispersed randomly throughout the cytoplasm and concentrated prominently on the nucleus margins after treatment with *Lonicera japonica* extracts and light (Figure 3). The effect of rottlerin on the photoactivated *Lonicera japonica* extract-induced changes in CH27 cell tubulin was also examined. As shown in Figure 3, rottlerin (10 *μ*M) significantly inhibited the photoactivated *Lonicera japonica* extract-induced changes in the organization and distribution of tubulin (Figure 3).

### 3.4. Effects of Rottlerin on the Photoactivated *Lonicera japonica* Extract-Induced Changes in Protein Expression of Cytoskeleton-Related Proteins in CH27 Cells

**Figure 4:**
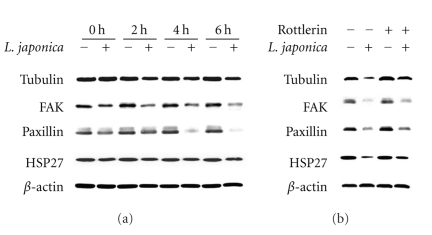
(a) Effects of photoactivated *Lonicera japonica* extracts on the expression of cytoskeleton-related proteins in CH27 cells. Cells were incubated with 0.1% ethanol or 100 *μ*g/mL* Lonicera japonica* extracts for 0, 2, 4, and 6 h and then irradiated with 0.8 J/cm^2^ fluence dose. (b) The effect of rottlerin on the photoactivated *Lonicera japonica* extract-induced changes in cytoskeleton-related proteins expression of CH27 cells. Cells were pretreated with 10 *μ*M rottlerin for 1 h and then treated with 0.1% ethanol or 100 *μ*g/mL* Lonicera japonica* extracts for 4 h and 0.8 J/cm^2^ fluence dose. After irradiation, cell lysates were analyzed by 5% (FAK), 9% (paxillin), 10% (tubulin), and 13% (HSP27) SDS-PAGE and then probed with primary antibodies as described in Section 2. −: control cells; +: *Lonicera japonica* extract- or rottlerin-treated cells. Results are representative of three independent experiments.

Results described above suggested that the photoactivated *Lonicera japonica *extract-induced apoptosis is mediated by change in distribution of cytoskeleton. Therefore, we focused the attention on the expression of cytoskeleton-related proteins, such as tubulin, focal adhesion kinase (FAK), paxillin, and heat shock protein (HSP27). As shown in Figure 4(a), the expression of tubulin, FAK, paxillin, and HSP27 was significantly decreased after treatment with 100 *μ*g/mL *Lonicera japonica* and irradiation with 0.8 J/cm^2^ light dose. The photoactivated *Lonicera japonica*-induced decrease in tubulin, FAK, paxillin, and HSP27 protein levels was partly reversed by pretreatment with 10 *μ*M rottlerin (Figure 4(b)).

### 3.5. Effects of Rottlerin on the Photoactivated *Lonicera japonica* Extract-Induced Changes in Mitochondrial Function in CH27 Cells

**Figure 5:**
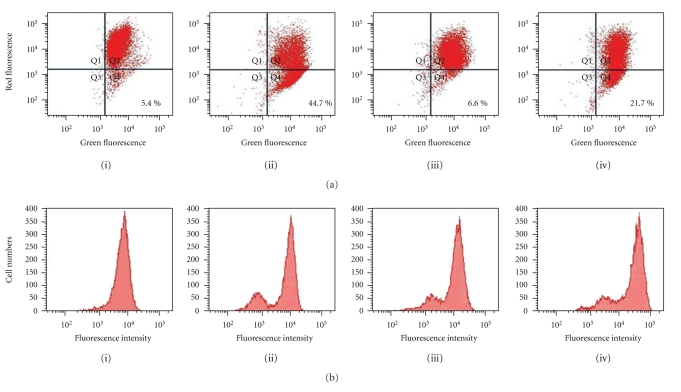
Effects of rottlerin on the photoactivated *Lonicera japonica* extract-induced disruption of mitochondrial function in CH27 cells. Cells were incubated with vehicle alone or 100 *μ*g/mL* Lonicera japonica* extracts for 4 h and then irradiated with 0.8 J/cm^2^ fluence dose. In rottlerin treatment, cells were pretreated with a final concentration of 10 *μ*M rottlerin for 1 h. (a) The fluorescent cation dye JC-1 was used to determine the mitochondrial membrane potential. After irradiation, the cells were harvested and stained with 2 *μ*M JC-1 for 15 min. The mitochondrial depolarization patterns of the CH27 cells were measured by flow cytometry. The sum of the percentage of Q1, Q2, Q3, and Q4 is 100%. (b) The effect of rottlerin on the photoactivated *Lonicera japonica* extract-induced opening of mitochondrial permeability transition (MPT) pore in CH27 cells. Before treatment with *Lonicera japonica* extracts and light, cells were loaded with 1 *μ*M calcein AM for 30 min in DMEM medium containing 1 mM CoCl_2_. After irradiation, the cells were harvested and then analyzed by flow cytometry for loss of fluorescence intensity due to efflux of the dye. (i) control cells; (ii)* Lonicera japonica* extract-treated cells; (iii) rottlerin-treated cells; (iv) cells were pretreated with rottlerin and then *Lonicera japonica* extracts. Results are representative of three independent experiments.

In the experiments following, the effect of photoactivated *Lonicera japonica* extracts on mitochondrial membrane potential of CH27 cells was examined. The mitochondrial membrane potential was investigated with the fluorescent probe JC-1. After cells were treated with 100 *μ*g/mL *Lonicera japonica* extracts for 4 h and then irradiated with 0.8 J/cm^2^ fluence dose, a remarkable attenuation of mitochondrial membrane potential occurred compared to the control cells (Figure 5(a)). As shown in Figure 5(a), photoactivated *Lonicera japonica* extracts induced a marked decrease in red fluorescence intensity compared to those in control cells. The photoactivated *Lonicera japonica*-induced disruption of mitochondrial membrane potential was partly blocked by pretreatment with 10 *μ*M rottlerin (Figure 5(a)). To further confirm the possible involvement of mitochondrial permeability transition (MPT) pores in the process of the photoactivated *Lonicera japonica* extract-induced apoptosis, we measured the opening of MPT pore in intact cells by flow cytometry. As shown in Figure 5(b), treatment with 100 *μ*g/mL *Lonicera japonica* extracts for 4 h and irradiation with 0.8 J/cm^2^ light dose resulted in the formation of a group of cells with lower calcein fluorescence intensity due to the opening of MPT pores. Rottlerin alone also showed the same phenomenon as the *Lonicera japonica* extract-sensitized/irradiated CH27 cells (Figure 5(b)). The photoactivated *Lonicera japonica* extract-induced the opening of MPT pore was not enhanced by pretreatment with 10 *μ*M rottlerin (Figure 5(b)). Based on the above data, mitochondrial function was involved in the photoactivated *Lonicera japonica* extract-induced CH27 cell death.

## 4. Discussion

Many factors such as cytoskeleton-related proteins, protein kinase C (PKC) family, Rho family, and mitogen-activated protein kinase (MAP kinase) family have been demonstrated to be involved in apoptosis. PKC family, which functions through serine/threonine kinase activity, is involved in signal transduction pathways necessary for cell proliferation, differentiation, and apoptosis [12, 13]. PKC*δ* has been reported as a proapoptotic response to various apoptotic stimuli, such as radiation and chemotherapeutic agents [14, 15], whereas PKC*α* and PKC*ε* have been mainly associated with antiapoptotic effects [16, 17]. A familiar signaling pathway is the MAP kinase cascades which play a central role in the cellular response to various extracellular stimuli [18, 19]. Three subgroups of MAPKs are known: extracellular signal-regulated kinases (ERK1/2), jun N-terminal kinase/stress-activated protein kinase (JNK/SAPK), and p38. Activation of MAP kinase members has been implicated in the regulation of apoptotic cell death [18, 20]. Furthermore, PKC is known to activate ERK1/2 pathway by stimulating the Ras-Raf-MAP kinase/ERK kinase-ERK pathway [21, 22]. Mhaidat et al. (2007) have suggested that PKC*ε* is acting upstream of docetaxel-induced ERK1/2 activation [23]. Ras, a small GTPase protein, is an important regulator of cell growth in all eukaryotic cells. Ras mediates its effects on cell proliferation and apoptosis in part by activation of the Raf/MEK/ERK cascade of protein kinases [24, 25]. The Rho family members are small GTPases and key regulators of actin dynamics, together with the related proteins Rho and Cdc42, coordinating formation of stress fibers, focal adhesions, lamelipodia, and filopodia and thus regulating overall cellular movement and cell morphology [26, 27]. To identify the overall signaling pathways involved in photoactivated *Lonicera japonica*-induced CH27 cell apoptosis, we examined most proteins expression of apoptotic signaling pathways such as PKC, Rho, and MAP kinase family members in this study.

In this study, there was a significant decrease in protein expression of PKC, Rho, and MAP kinase family members after treatment with *Lonicera japonica* extracts and light. The results of PKC protein expression are consistent with those of other studies reporting that the overexpression and activation of PKC*α* and PKC*ε* have been mainly associated with inhibition of apoptosis [16, 17]. Proteolytic cleavage of PKC*δ* by caspase-3 at the V3 (hinge) domain of the enzyme releases a catalytically active fragment of approximately 40 kDa. It is generally believed that proteolytic activation of PKC*δ* is responsible for apoptotic execution [14, 15]. However, this study could not detect the presence of PKC*δ* catalytic fragment after* Lonicera japonica* extracts with irradiation. In this study, PKC*ε* is clearly identified as a doublet with molecular masses about 90 kDa. PKC*ε* has often been detected as a doublet, but this has been rarely commented on [28, 29]. It has also been demonstrated that PKC*ε* purified from rat brain migrates as two distinct bands with molecular masses of 93 kDa and 96 kDa; on phosphatase treatment, these are reduced to a single 90 kDa species [30]. Rottlerin reversed the photoactivated *Lonicera japonica* extract-induced change in PKC protein expression. Based on the above data, it seemed reasonable to surmise that prevention of CH27 cells from the photoactivated *Lonicera japonica* extract-induced cell death of rottlerin is associated with the activity of PKC family. 

In this study, it was also found that rottlerin reversed the protein expression of many kinases of prosurvival signaling pathway, such as Ras, ERK, and p38, during the photoactivated *Lonicera japonica* extract-induced cell death. It is noteworthy that Western blot analysis of the protein expression of Ras exhibits two bands (21 and 22 kDa) in this study. The amount of 21 kDa of Ras significantly decreased after treatment of CH27 cells with *Lonicera japonica *extracts and light, whereas the protein expression of 22 kDa of Ras was increased. It is wellknown that Ras prenylation in which a lipid anchor is added to the C-terminus of proteins to allow membrane association plays a central role in the development of cancer. When the prenylation of Ras was blocked, a shift to a higher mobility band was seen on SDS-PAGE [31, 32]. The protein bands of 21 and 22 kDa of Ras may be its prenylated and unprenylated status, respectively. Therefore, we guess that photoactivated *Lonicera japonica* extracts induced CH27 cell death through blocking the prenylation of Ras. 

Several reports have indicated that cytoskeleton may play a primary role in the initiation phase of apoptosis in certain circumstances [4, 5]. Microtubules and actin microfilaments are the major proteins of cytoskeleton and both regulate cell shape. The involvement of cytoskeletal actin in the photoactivated *Lonicera japonica* extract-induced apoptosis has been suggested by our previous study [3]. In this study, the photoactivated *Lonicera japonica* extract-induced disruption of actin microfilaments was prevented by pretreatment of CH27 cells with rottlerin. In addition to actin microfilaments, the microtubule also serves as key components of the cytoskeleton. This study demonstrated that rottlerin prevented the photoactivated *Lonicera japonica* extract-induced changes in the protein amount and distribution of tubulin. These data suggest that rottlerin can prevent the photoactivated *Lonicera japonica*-induced rearrangements of the actin microfilaments or microtubules and support the possibility that rottlerin, directly or indirectly, prevents the aggregation of cytoskeleton and stabilizes the CH27 cell cytoskeleton. HSP27 is a component of the large and heterogeneous group of chaperone proteins, and its main functions are prevention of aggregation of actin filament and inhibition of apoptosis [33]. It has been suggested that p38-mediated F-actin reorganization was associated with translocation of HSP27 from cytosolic to cytoskeletal fraction [34]. The photoactivated *Lonicera japonica*-induced decrease in p38 and HSP27 protein expression was significantly reversed by rottlerin in this study. This result suggested that the protein expression of p38 and HSP27, which may stabilize the actin cytoskeleton, may be one of the mechanisms for the protective effects of rottlerin against the photoactivated *Lonicera japonica*-induced CH27 cell death. In this study, the change in the protein expression of cytoskeleton-related proteins, such as tubulin, FAK, paxillin, and HSP27 was significantly observed in photosensitized *Lonicera japonica* extract-induced rapid cell death. Many intracellular events, including the assembly of focal adhesion, actin microfilament reorganization, and recruitment of many signaling molecules to the focal adhesions were involved in the change in cell shape [3–5]. This study also demonstrated that photoactivated *Lonicera japonica* extracts induced a marked morphological change, where normally flat cells became round in shape and detached from the extracellular matrix. This finding suggested that the photoactivated *Lonicera japonica* extract-induced cell death and changes in cell morphology are mediated in part through its effect on cytoskeleton in CH27 cells. The present study also demonstrated that the photoactivated *Lonicera japonica* extract-induced CH27 cell death and changes in cell morphology were blocked by pretreatment with 10 *μ*M rottlerin.

Many evidences indicated the potential role of actin cytoskeleton in facilitating the mitochondrial recruitment of various proapoptotic proteins, such as cytochrome *c* or apoptosis-inducing factor, to the cytosol to initiate apoptosis [5–7]. The mitochondrial permeability transition (MPT) pore is characterized by opening of the permeability transition pore in the inner mitochondrial membrane, which results in an increase in permeability of this membrane to protons, ions, and small molecular weight solutes [35]. Therefore, the opening of MPT pore is a critical step in the process that allows mitochondria to release cytochrome *c* or apoptosis-inducing factor into the cytoplasm [36, 37]. This increased permeability is also considered to lead to a collapse of the mitochondrial membrane potential. We demonstrated that exposing the CH27 cells to *Lonicera japonica* extracts and light resulted in a significant induce of the opening of MPT pores accompanied by a disruption of mitochondrial membrane potential in CH27 cells. Therefore, we hypothesize that the photoactivated *Lonicera japonica* extract-mediated apoptosis occurs through the disruption of mitochondrial function. We also demonstrated that the photoactivated *Lonicera japonica* extract-induced disruption of mitochondrial membrane potential was significantly blocked by pretreatment with rottlerin. Rottlerin had a slight effect on the photoactivated *Lonicera japonica* extract-induced opening of MPT pores. Based on the above data, the opening of MPT pore may be involved in photoactivated *Lonicera japonica* extract-induced CH27 cell apoptosis, but not a major factor. However, pretreatment of CH27 cells with rottlerin could prevent the photoactivated *Lonicera japonica* extract-induced disruption of mitochondrial membrane potential, providing further support for the role of PKC*δ* as a mediating factor of mitochondria during the photoactivated *Lonicera japonica* extract-induced cell apoptosis. 

This study indicated that many apoptotic signaling pathways, such as Rho family, PKC family, MAP kinase family, and mitochondria-mediated apoptotic pathway, were triggered by *Lonicera japonica* extracts and irradiation in CH27 cells. Our findings suggested that promotion of the cytoskeleton-related signaling cascade following rottlerin by upregulation of cytoskeleton-related mediators (p38, HSP27, FAK, paxillin and tubulin) and molecules of downstream of F-actin (mitochondria-mediated apoptosis pathway) reduce CH27 cell death, indicating that cytoskeleton is the potential target in the photoactivated *Lonicera japonica* extract-induced CH27 cell apoptosis.
